# Editorial of the Special Issue: Extracellular Vesicles and Exosomes as Therapeutic Agents

**DOI:** 10.3390/biomedicines13092234

**Published:** 2025-09-11

**Authors:** David J. Rademacher

**Affiliations:** Department of Microbiology and Immunology and Core Microscopy Facility, Stritch School of Medicine, Loyola University Chicago, Maywood, IL 60153, USA; drademacher@luc.edu; Tel.: +1-414-628-9063

## 1. Introduction

Membrane-enclosed particles, known as extracellular vesicles (EVs), are ubiquitously present in organisms, including animals, plants, and microorganisms [1,2]. EVs are classified according to their size and origin as apoptotic bodies, microvesicles, or exosomes (EXs) [1,2,3]. EXs, the most extensively studied EVs, are enclosed in a single phospholipid bilayer and are secreted by all cell types [1,3,4,5]. EXs are usually 30 to 150 nm in diameter and are formed by fusion of the multivesicular body and the inward invagination of the endosomal membrane [1,3,4,5]. EXs are present in body fluids such as saliva, amniotic fluid, breast milk, urine, and plasma [6]. They are essential for intercellular communication because they change the functions of recipient cells by delivering a variety of biologically active cargo, including proteins, lipids, and nucleic acids [7,8].

The use of EXs as a delivery strategy for therapeutic cargo has become increasingly popular, in part, because they overcome many of the challenges encountered by other approaches. EXs exhibit a high degree of stability and biocompatibility with minimal immunogenicity [9]. The delivery-to-target efficiency is higher for EXs than conventional approaches because they can traverse biological barriers, such as the blood-brain barrier (BBB) [10,11]. EXs can selectively target tissues or cells and reduce off-target effects because of their inherent tropism based on their cells of origin [12]. EXs provide a protective environment for the encapsulated cargo, safeguarding it from degradation [13]. Furthermore, because EXs are the primary way that stem cells exert their biological effects, the use of stem cell-derived EXs preserves the therapeutic potential of stem cells while avoiding any potential harm from the cells [14,15]. Importantly, systemic administration of EXs derived from a variety of cell types often results in their primary accumulation in the liver and spleen, with less than 1% of that administered reaching the desired target [12,16]. Consequently, several engineering approaches have been developed to improve targeting and increase the accumulation of EXs in the target tissue and/or cells.

## 2. Recent Developments in the Use of Extracellular Vesicles and Exosomes as Therapeutic Delivery Systems

### 2.1. Genetic Engineering of EXs

Genetic engineering has been used to modify exosomal surface proteins to better target EXs to tissues and cell types of interest, since even slight variations in exosomal tetraspanin complexes have a significant impact on target cell selection. Transfecting genes expressing a targeting moiety with exosomal membrane components is one method of genetically altering EX-producing cells. Through the natural EX biogenesis process, the cells transfected with these vectors produce surface-modified EXs that express the targeting moieties. EXs produced from genetically engineered cells stably display the introduced targeting moiety on their surface [17]. For example, Wang and colleagues [18] genetically engineered stem cell-derived EXs loaded with a ferroptosis inhibitor to target M2 microglia. The genetically engineered EXs exhibited M2 microglia targeting specificity in vitro and in vivo, inhibited ferroptosis in M2 microglia, and improved neurological function in ischemic stroke-affected mice [18]. A fusion protein, consisting of rabies virus glycoprotein (RVG), a protein that binds nicotinic acetylcholine receptors, and lysosomal membrane-associated protein 2B, an exosomal membrane protein, was transfected into 293T cells. The cells produced EXs with RVG embedded in the exosomal membrane [19]. As neurons and glia express receptors that bind to RVG on their cell surface, systemic administration of RVG-expressing EXs resulted in a two-fold increase in brain EX accumulation compared to systemic administration of non-engineered EXs [19].

### 2.2. Chemical Modifications of EXs

To make therapeutic EXs more targetable, their surface can be directly engineered through chemical modifications. One such approach is click chemistry. Click chemistry utilizes covalent interactions between an alkyne and an azide residue to form a stable triazole linkage, which can be applied to attach targeting moieties on the surface of EXs [20,21]. For example, Jia and colleagues [22] used click chemistry to conjugate the RGERPPR peptide (RGE) to the exosomal membrane. The engineered EXs delivered therapeutic cargo to intracranial tumors because RGE binds specifically to neuropilin-1 (NRP-1), which is overexpressed in glioma cells and is either not expressed at all or expressed at a low level in normal neurons and other tissues [22]. Chen and coworkers [23] used click chemistry to attach a targeting peptide to the surface of macrophage-derived EXs. These engineered EXs targeted bone tissue and released anti-microbial peptides, thereby relieving osteomyelitis without adverse effects in a rat model [23].

### 2.3. Combinatorial Approaches

#### Hybrid Nanoparticles

As an alternative to genetic engineering of parent cells or EXs and chemical modification of EXs, targeting ligands conjugated to polyethylene glycol (PEG) have been used to produce EXs in which the targeting ligand is expressed in the exosomal membrane. For example, nanobodies specific for epidermal growth factor receptor (EGFR), upregulated in a variety of solid tumors, were conjugated to phospholipid-PEG derivatives to produce nanobody-PEG-micelles [24]. Nanobody-PEG-micelles were expressed in the exosomal membrane when they were incubated with EXs; this had no effect on the size distribution, protein composition, or morphology of EXs. The PEGylated and targeted EXs increased the binding to tumor cells overexpressing EGFR and were retained in the circulation for six times longer than unmodified EXs [24].

Diacyl-chain phospholipids self-assemble in aqueous solutions to form spherical vesicles called liposomes [25]. Liposomes expressing tLyp-1 (CGNKRTR), which has a high affinity to NRP-1 to target glioma cells, were created and fused with EXs, which have an enhanced ability to cross the BBB, in part because of the expression of transferrin receptors on the EX’s surface, to create hybrid liposome-EX nanovesicles with an enhanced ability to cross the BBB and target brain tumors [26,27]. Compared with liposomes and EXs, the hybrid nanovesicles demonstrated an increased ability to cross the BBB and accumulate in glioma in mice [26].

In a fascinating study, adipose-derived mesenchymal stem cell (ADSC) EXs and gelatine nanoparticle (GNP) hydrogel combined to form a stable meshwork because of the opposing charges of the two substances. In a rat skull defect model, the skull bone formation produced by the GNP hydrogel-ADSC EX mixture was substantially greater than that of the GNP- and sham-treated groups [28].

Liu and colleagues [29] created a nanocomplex, referred to as REXO-C/ANP/S, which was comprised of a core and a shell. The core, referred to as C/ANP/S, was comprised of: (a) curcumin, a small molecule that disrupts α-synuclein aggregates; (b) small interfering RNA targeting *SNCA* to downregulate α-synuclein synthesis; and (c) a reactive oxygen species (ROS)-responsive polymer, poly(2-(dimethylamino)ethyl acrylate, which encapsulates curcumin and enables ROS-responsive drug release. The shell, termed REXO, was comprised of: (a) immature dendritic cell-derived EXs; and (b) RVG peptide, a targeting peptide embedded in the exosomal membrane to enhance delivery across the BBB and target neurons. In a mouse model of Parkinson’s disease, the REXO-C/ANP/S nanoparticles decreased α-synuclein aggregates in diseased neurons [29].

### 2.4. Large-Scale EX Production Approaches

The quality and quantity of secreted EXs are reduced in conventional two-dimensional, static cultures, as these cultures cannot replicate the in vivo environment, including fluidic stimuli [30]. To overcome these limitations, Huang and coworkers [31] developed a method of culturing genetically engineered mesenchymal stromal cells to increase the production of EXs rich in hepatocyte growth factor (HGF) for improved wound healing. Two structured polydimethylsiloxane layers were used to construct a microfluidic chip, which was a crucial part of their culturing technique. The top layer had herringbone grooves, and the bottom layer had a micropillar array. By disrupting the laminar flow, these structures produced a turbulent vortex that improved waste removal and nutrient delivery. In addition, the bottom micropillar layer provided a large number of cell adhesion sites. The EX yield obtained from cells cultured on the microfluidic chip was ~14 times that obtained from cells cultured in a conventional, two-dimensional static cell culture flask. Furthermore, the EXs derived from microfluidic chip-cultured cells were abundant in HGF and aided in wound healing [31].

Contrary to conventional, static cell culture conditions, a flat-plate bioreactor was used to increase EX production. The chamber in the flat-plate bioreactor was seeded with cells. The culture medium was delivered to the chamber via a peristaltic pump, which produced laminar flow conditions. The flow introduced shear stress to the cells, which opened calcium channels, leading to increased calcium influx [32]. Prior studies have shown that increasing intracellular calcium concentration increases EX yield [33]. A closed-loop system was used to collect and recirculate the culture medium, which ensured a continuous exchange of oxygen and nutrients while eliminating waste. The bioreactor produced ~7 times more EXs than static culture conditions, while preserving the quality and therapeutic efficacy of the EXs [32].

### 2.5. Milk and Plant EXs

Milk- and plant-derived EXs have gained popularity recently despite advancements in the mass production of EXs derived from cultured cells. Milk is a particularly dense source of EXs, with concentrations in milk exceeding 10^12^ EXs per milliliter [34]. Due to their stability, scalability, ability to cross biological barriers, and between-species biocompatibility, recent research has focused on the development of milk EXs as a drug delivery platform. Milk EXs are non-immunologic oral delivery vehicles for gene editing agents, peptides, small molecules, and bioactive RNA cargo [35,36,37]. However, the isolation and characterization of milk EXs is complicated by the fact that milk contains a variety of nanoparticles besides EXs [38]. Continued research is crucial to harness the full potential of milk EXs in clinical and therapeutic contexts.

Similar to milk-derived EXs, plant-derived EXs represent a potentially rich source of useful EXs. Plant-derived EXs are thought to be used as a means of intercellular communication and immune regulation to protect plants from pathogenic attacks [39]. Notably, the size distribution, density, morphology, and surface electric charge of EXs derived from plants are comparable to those derived from mammals [40]. Like mammalian cell-derived EXs, they contain lipids, proteins, and RNAs, which reflect their plant sources, such as grapes, ginger, and apples [41,42,43]. The techniques used to isolate EXs from plants are not very different from those used to isolate EXs produced by cultured mammalian cells [44]. Plant EXs are safe [42] and their production is more scalable and cost-effective than EXs derived from mammalian cells [45]. Ongoing research is needed to establish standardized plant EX extraction protocols and evaluate long-term safety for clinical applications.

## 3. Contributions to This Special Issue

In this Special Issue, Mecocci and colleagues [46] described the anti-microbial and anti-inflammatory effects of cow colostrum-derived EXs in an in vitro model of neonatal calf diarrhea caused by *Escherichia coli* infection. Their results present the fascinating prospect that colostrum-derived EXs may provide an alternative to antibiotics in combating drug-resistant bacterial infections. Schepici and coworkers [47] provided a comprehensive review of the regenerative effects of mesenchymal stromal/stem cell-derived EXs in spinal cord injury and a summary of the preclinical studies on their therapeutic potential. Mohammad and colleagues [48] examined the effects of exercise-induced EXs on trophoblasts in vitro, highlighting their potential role in maternal–fetal communication during pregnancy. Rademacher [49] described the dual role of EXs in Parkinson’s disease, as both a contributor to disease progression and a potential therapeutic tool. In Parkinson’s disease, EXs act as mediators of disease progression by spreading toxic proteins and promoting neuroinflammation. However, their natural role in intercellular communication and ability to deliver therapeutic agents make them a promising tool for developing next-generation therapies [49]. Wang and coworkers [50] investigated the role of EXs derived from osteogenic-differentiated human bone marrow-derived mesenchymal cells (hBMSCs) in the rescue of the osteogenic ability of hBMSCs impaired by hypoxia. They discovered that EXs derived from hBMSCs cultured in normoxia restored the osteogenic capacity of hBMSCs that had been compromised by hypoxia, offering a possible treatment approach for bone regeneration in hypoxic conditions [50]. The literature on the effects of physical cues on stem cell-derived EXs to treat peripheral neuropathy was reviewed by Berry and colleagues [51]. They concluded that physical cues such as electrical stimulation, mechanical agitation, and conductive materials greatly improved the production, quality, and therapeutic potential of stem cell-derived EXs. These improvements make EXs more effective in treating peripheral neuropathy [51]. The efficiency and quality of artificial EXs produced by T cells using two different induction techniques—cytochalasin B, a chemical inducer, and ultrasonication, a physical inducer—were compared by Zmievskaya and colleagues [52]. Garaeva and colleagues [53] discovered that EXs loaded with exogenous, recombinant heat shock protein 70 derived from grapefruit triggered an anti-tumor immune response in both in vitro and in vivo models of colorectal cancer, highlighting the potential of plant-derived EXs as effective therapeutic cargo delivery vehicles. Jones and coworkers [54] determined the efficacy of lyophilized EXs derived from human adipose stem cells in promoting healing from traumatic brain injury, underlining their potential for storage at room temperature and clinical application. Finally, Salih and colleagues [55] investigated the expression of the microRNAs let-7a-5p and miR-21-3p in EXs derived from the serum of non-small-cell lung cancer patients, highlighting their potential as diagnostic biomarkers.

## 4. Future Research

High production costs, a high level of technical expertise, and challenges differentiating genetically engineered EXs from naturally occurring EXs in biological fluids are some of the drawbacks of genetically engineered EXs, despite their many benefits such as improved targeting precision, effective therapeutic delivery, and decreased off-target effects. Further research and optimization are needed to make them more feasible for clinical applications. Chemically modified EXs offer several advantages, including enhanced targeting, versatility, retention of the natural properties of the EXs, and the ability to cross biological barriers. The technical difficulties of both covalent and non-covalent EX modifications, the possible loss of native protein function in the EX membrane, and the difficulty in scaling up chemical modification techniques to large-scale production are the drawbacks of chemical modification of EXs. Future studies should focus on addressing these issues. Potential disruption of the exosomal membrane during production, the possibility of an immunological reaction, and regulatory ambiguity surrounding their classification—which makes approval more difficult—are some of the difficulties with hybrid nanoparticles. Clear regulatory frameworks, scalable production methods, and improved engineering techniques are essential to tackle these challenges. Future developments in isolation technologies, engineering methods, and standardization protocols are required to enable scalable EX production for clinical applications, even though recent advancements in the large-scale production of EXs, such as the use of microfluidic chips and bioreactors, are encouraging. Despite the fact that milk EXs are non-immunologic oral delivery vehicles for a range of therapeutic substances, a major obstacle is the transfer of isolation techniques from the laboratory to the industrial production process. In addition, there are no detailed pharmacokinetic studies, and the complex composition of milk makes it difficult to isolate high-purity milk EXs. To address these issues, improvements in isolation techniques, scalable production methods, and thorough safety and efficacy studies are needed. Despite the fact that plant EXs are safe [42] and can be produced more cheaply and efficiently than EXs produced by mammalian cells [45], preclinical and clinical research are required to confirm their therapeutic efficacy, optimize dosages, and assess long-term effects before they can be used successfully in clinical settings.

## Data Availability

Not applicable.
